# P38 kinase in gastrointestinal cancers

**DOI:** 10.1038/s41417-023-00622-1

**Published:** 2023-05-29

**Authors:** Thuy Phan, Xu Hannah Zhang, Steven Rosen, Laleh G. Melstrom

**Affiliations:** 1 Department of Surgery, City of Hope Medical Center, Duarte, CA USA; 2 Department of Hematology, City of Hope Medical Center, Duarte, CA USA

**Keywords:** Cancer, Drug discovery, Medical research

## Abstract

Gastrointestinal cancers are a leading cause of cancer morbidity and mortality worldwide with 4.2 million new cases and 3.2 million deaths estimated in 2020. Despite the advances in primary and adjuvant therapies, patients still develop distant metastases and require novel therapies. Mitogen‑activated protein kinase (MAPK) cascades are crucial signaling pathways that regulate many cellular processes, including proliferation, differentiation, apoptosis, stress responses and cancer development. p38 Mitogen Activated Protein Kinases (p38 MAPKs) includes four isoforms: p38α (MAPK14), p38β (MAPK11), p38γ (MAPK12), and p38δ (MAPK13). p38 MAPK was first identified as a stress response protein kinase that phosphorylates different transcriptional factors. Dysregulation of p38 pathways, in particular p38γ, are associated with cancer development, metastasis, autophagy and tumor microenvironment. In this article, we provide an overview of p38 and p38γ with respect to gastrointestinal cancers. Furthermore, targeting p38γ is also discussed as a potential therapy for gastrointestinal cancers.

## Introduction

Gastrointestinal (GI) cancers of the colon, stomach, liver, esophagus, and pancreas impacted an estimated 4.2 million patients (22%) and resulted in 3.2 million deaths (32%) in 2020 and together are a leading cause of cancer morbidity and mortality worldwide [1]. Current treatments for GI cancers involve multiples therapies including surgery, radiation, chemotherapy or a combination thereof. Based on the particular malignancy, radiation therapy is one of the treatments for GI cancer that can be applied before or after surgery. Chemotherapy is also combined with radiation to enhance tumor sensitization. Immunotherapy for GI cancers includes checkpoint inhibitors (PD-1, PD-L1 and CTLA-4), vaccine therapies (peptide, protein, whole tumor cells, or dendritic cell-based vaccines), cytokines (interferon-γ, interleukin-2, IL-10, or GM-CSF) and adoptive T cell transfer [2]. Until now, surgical resection remains the most common treatment for patients with colorectal [3], gastric [4], and esophageal cancers [5]. Despite the advances in primary and adjuvant therapies, patients can still develop metastases and resistance to systemic therapy. Therefore, new therapeutic strategies and novel targets are urgently in need to improve the survival.

The mitogen-activated protein kinases (MAPK) pathways consist of three distinct kinases that play crucial roles in cell signaling. When exposed to extracellular and intracellular signals, MAPKKKs are activated and facilitate the direct phosphorylation of MAPKKs, leading to their activation[6]. Subsequently, MAPKKs phosphorylate and activate MAPKs [7]. MAPKs respond to a diverse range of stimuli, such as hormones, cytokines, growth factors, endogenous stress, and environmental signals [8]. Extracellular signal-regulated kinases (ERK), c-Jun NH2-terminal kinase (JNK), and p38 are the three well-known MAPKs that play a role in carcinogenesis. Among these, the ERK pathway is predominantly triggered by growth factors like epidermal growth factor, as well as hormones and proinflammatory stimuli. On the other hand, the JNK and p38 pathways are activated by different stress-inducing stimuli, such as ultraviolet radiation, reactive oxygen species (ROS), as well as inflammatory cytokines like tumor necrosis factor (TNF)-α and interleukin (IL)-1β [9]. MAPKs are involved in regulating a range of cellular processes linked to the development of cancer, including proliferation, differentiation, apoptosis, inflammation, and immunity. Abnormal MAPK signaling may result in excessive or unregulated cell proliferation, as well as resistance to apoptosis[10].

## p38 pathways

**Fig. 1:**
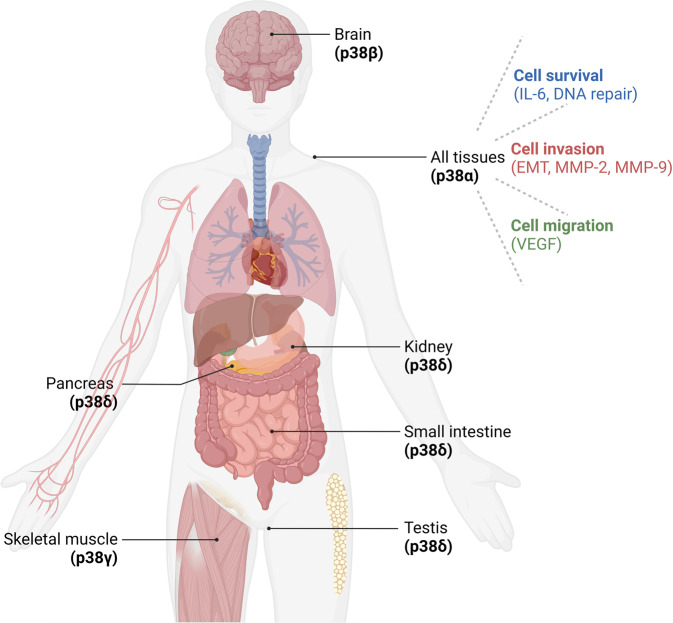
p38 distribution and function. The p38 MAPKs include 4 isoforms (α, β, γ, and δ) ubiquitously expressed with different levels of expression in various organs. p38 MAPK supports cell survival via anti-apoptotic inflammatory signals interleukin-6 (IL-6) and enable DNA-repair after chemotherapy which results in drug-resistance in cancer cells. Upregulation of p38 MAPK promotes cell invasion by inducing epithelial to mesenchymal transdifferentiation (EMT), matrix metalloproteinases MMP-2 and MMP-9. Activated p38 signaling also increases cell migration via Vascular Endothelial Growth Factor (VEGF).

The p38 MAPK family was first identified in studies of endotoxin-induced cytokine expression [11]. P38 is involved in inflammation, cell growth, cell differentiation, cell death, and the cell cycle [12]. There are four p38 isoforms including p38α (MAPK14), p38β (MAPK11), p38γ (SAPK3, ERK6 orMAPK12), and p38δ (MAPK13) with an overall sequence homology >60% and an identity within the kinase domains >90% [13]. The four p38 MAPK isoforms are ubiquitously expressed with different levels of expression in various tissues (Fig. 1). For example p38α is expressed in all cell types and tissues; p38β is predominant in the brain; p38γ is found in skeletal muscle and p38δ is mainly expressed in the testis, pancreas, kidney and small intestine [14]. Despite the high level of sequence homology of the four isoforms, they interact with different downstream effectors such as: MK2 (MAPK-activating protein kinase 2), PRAK (p38-related/activated protein kinase), ATF-2 (activating transcription factor-2), MEF2 (myocyte enhancement factor 2) and c-Jun and upstream MAPK kinase activators (including MKK4, MKK3 and MKK6) [15]. Phosphorylated p38 MAPK can activate a wide range of stimuli, such as transcription factors, protein kinases, cytoplasmic substrates and nuclear substrates [16]. The downstream events of these p-p38 MAPK have cell-specific consequences including regulation of RNA splicing, cytokine production, inflammatory response, apoptosis, cell-cycle arrest and cell differentiation [16]. Activated p38α has been shown to downregulate cyclins, upregulate cyclin-dependent kinase (CDK) inhibitors, modulate the tumor suppressor p53 at the G1/S and the G2/M phases and induce apoptosis. This reduces cell proliferation in primary cells (cardiomyocytes, hepatocytes, fibroblasts, hematopoietic cells, and airway epithelium). In contrast, p38α has also been shown to support cell survival via anti-apoptotic inflammatory signals interleukin-6 (IL-6) and enable DNA-repair after chemotherapy which results in drug-resistance in cancer cells [17]. Moreover, upregulation of p38 MAPK promotes cell invasion by inducing epithelial to mesenchymal transdifferentiation (EMT) [18]. Downregulation of p38 MAPK leads to suppressed expression and activity of matrix metalloproteinases MMP-2 and MMP-9. This evidence supports the role of p38 in facilitating cancer cell invasion [19]. Activated p38 signaling also increases cell migration via Vascular Endothelial Growth Factor (VEGF) expression promoting actin rearrangement [20]. The studies noted above demonstrate a duality in the role of p38 MAPK signaling as either an oncogene or tumor suppressor in many types of tumors.

In resting cells, p38α/β are mainly found in the cytoplasm, with certain molecules undergoing phosphorylation in response to stimulation [21]. Whether phosphorylated or not, p38α/β form a complex with either a dimer of Imp7/3 or Imp9/3, which transport them to the nuclear pores. While Imp3 remains outside, Imp7 or Imp9 accompany the p38α/β into the nucleus. Once inside, p38α/β disassociate from the importins and proceed to phosphorylate their substrates. Finally, they are exported back to the cytoplasm. Upon activation in myoblasts, the p38 signaling pathway phosphorylates BAF60c, facilitating the recruitment of the SWI-SNF chromatin-remodeling complex to muscle-specific regions. This, in turn, promotes the activation of gene expression [22].

**Fig. 2:**
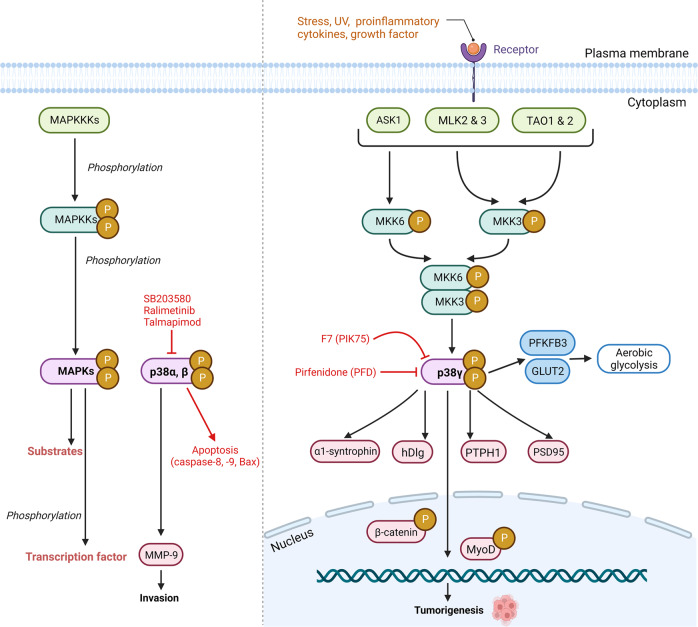
Schematic representation of p38γ MAPK signal transduction pathway. A wide variety of stimuli including cellular stresses, UV, proinflammatory cytokines, growth factors can activate p38γ MAPK. These lead to the initiation of a three-step MAPK phosphorylation cascade (MAPKKK, MAPKK, and MAPK). Firstly, MAPKKKs (ASK1, MLK1&2, and TAO1&2) phosphorylate the p38 MAPK-specific MAPKKs MKK6 and MKK3, respectively. These subsequently phosphorylate p38γ MAPK. The phosphorylated p38γ can activate the downstream substrates such as α-syntrophin, hDlg (human disc large), PTPH1 (protein tyrosine phosphatase H1), PSD95 (post-synapse density 95), and transcription factor MyoD, which trigger cellular responses. ASK1 (apoptosis signal-regulating kinase-1), MLKs (mixed-lineage kinases), TAO (thousand-and-one amino acid).

Among the MAPK family, p38γ is the only isoform that possesses a short C-terminal sequence (-KETXL) capable of binding to PDZ domains. When exposed to stress, p38γ can phosphorylate and regulate the activity of various PDZ-domain containing proteins involved in different signaling pathways. These include α1-syntrophin [23], SAP (synapse-associated protein) 90/PSD (post-synapse density) 95 [24], the scaffold protein SAP97/hDlg, and the protein tyrosine phosphatase PTPH1 [25] (Fig. 2). Interestingly, p38γ can also directly phosphorylate the transcription factor MyoD without interaction with the PDZ domain and subsequently suppress its activity [26]. In regard to tumorigenesis, p38γ binding to PTPH1 via the PDZ domain increases Ras transformation and promotes colon cancer development [25].

This review will aim to assess the role of p38 in cancers of the gastrointestinal tract.

## p38 in gastrointestinal cancers

### Esophageal cancer

In esophageal squamous cell carcinoma (ESCC), most publications regarding the p38 MAPK pathway in carcinogenesis have been focused on exploring the role of the p38α isoform. There is limited published data on other p38 isoforms (β, γ, and δ). Overall p38 MAPK expression has been found to be significantly higher in ESCC compared to normal esophageal tissue [27]. Zheng et al. found a significant association between p38γ expression and clinical stage, lymph nodes metastases, and tumor volume in ESCC [28]. This suggested p38γ may serve as a metastasis-associated gene in ESCC. Moreover, p38γ can promote cell motility and growth in ESCC cells in vitro. Knockdown of p38γ can prevent tumor progression in ESCC tumor bearing nude mice. These findings indicate that p38γ plays an oncogenic role in ESCC and may be targeted for therapy [28].

## Gastric cancer

In the case of gastric cancer, Liu et al. demonstrated that cells pretreated with a combination of ERK1/2 and p38 inhibitors could enhance the anti-proliferative effects of 5-FU via suppressing the ERCC1 (Excision Repair Cross Complementation group 1) protein [29]. ERCC1 is upregulated in 5-FU-treated cells which may contribute to drug resistance. This study proposed that activated ERK1/2 and p38 kinases play a role in developing drug resistance via upregulating ERCC1 expression in gastric cancer [29].

Interferon gamma (IFN-γ) plays an important role in the innate and adaptive immune response as a cytokine with antitumor functions [30]. Zhao et al. reported that gastric cancer cells treated with IFN-γ underwent G1/S phase cell cycle arrest and had down-regulated p38γ expression [31]. This study suggested that IFN-γ may inhibit gastric cancer cell proliferation via regulating p38γ.

## Colorectal cancer

p38γ is markedly upregulated in colon cancer tissues compared to surrounding colon epithelial cells [32]. p38γ knockdown was found to inhibit tumor progression in a colitis-associated mouse model [33]. In the same study p38γ activated the β-catenin/Wnt pathways promoting CRC development [33]. This protumor role has been corroborated by others where p38γ knock down inhibited cell proliferation, migration, and induced apoptosis in colon cancer cells in vitro. Overexpression of p38γ promoted CRC cell progression. Loesch et al. found that overexpressed p38γ MAPK activated the transcription factor c-Jun, and then recruited p38γ as a cofactor to the matrix metalloproteinase 9 (MMP-9) promoter thus enhancing cell invasion [34]. These studies indicated p38γ as a potential therapeutic target for CRC.

## Pancreatic cancer

In pancreatic adenocarcinoma, p38 expression was associated with a shorter survival [35]. However, Zhong et al. reported that high expression of p38 MAPK was associated with improved survival [36]. All isoforms of p38 MAPK were found in various human pancreatic cancer cells in vitro [37]. The roles of the various p38 isoforms in pancreatic cancer are controversial. Tian et al. proved that inhibition of p38β decreased tumor progression while inhibition of p38α enhanced tumor formation in pancreatic cancer mouse models [37]. In addition, p38γ has been reported to promote PDAC development via KRAS signaling and aerobic glycolysis. Wang et al. indicated that KRAS mutation induced the expression and phosphorylation of p38γ, and subsequently enhanced PFKFB3 (6-Phosphofructo-2-Kinase/Fructose-2,6-Biphosphatase 3) and expression/phosphorylation of a glucose transporter GLUT2 (Glucose transporter 2). Moreover, PFKFB3 and GLUT2 depend on p38γ for aerobic glycolysis and tumor development [38]. This study proved that p38γ is crucial for the G1/S transition in cell cycle and proliferation. p38γ knockdown or p38γ inhibition in combination with a PFKFB3 inhibitor suppressed aerobic glycolysis and PDAC tumorigenesis [38]. The above data highlight the heterogenous role of p38 MAPKs isoforms and selective targeting will decide the promising effect for cancer treatment.

## Liver cancer

In hepatocellular carcinoma (HCC), high p38γ expression was inversely related to survival [39]. In vitro, p38γ knockdown suppressed proliferation and colony formation in HCC cell lines [39]. Iyoda et al. showed that mutant MKK6 (a common activator of four p38 MAPK isoforms) increases p38 pathway activation, caspase-3 activity and subsequently induces apoptosis in human hepatocellular carcinoma cell lines [40]. Tomás-Lobaet al. reported that p38γ shares a high sequence homology and substrate specificity with cyclin-dependent kinase (CDK)–cyclin protein which regulates cell division in cancer development [39]. In this study, p38γ expression correlated with the expression of fibrosis markers ACTA2 and COL1A, that support the progression of liver cancer. p38γ in human HCC biopsy samples were found to be overexpressed compared to healthy livers. The data involving HCC suggests the oncogenic role of p38γ in human liver tumors and p38γ as a promising target for liver cancer therapy.

Table 1 summarizes physiological function of p38γ and its impacts in human gastrointestinal cancers.

**Table 1:** Physiological function of p38γ and its impacts in human gastrointestinal cancers.

Type of cancer	Function	Impacts	References
Esophageal cancer	Oncogene	p38γ promotes the cell motility and growth in vitro. Knockdown of p38γ prevents the tumor formation in mice. p38γ expression is markedly associated with clinical stage, lymph nodes metastases, and tumor volume in ECSS tissues.	[28]
Gastric cancer	Oncogene	p38 and ERK1/2 inhibitors enhance the anti-proliferative effects of 5-FU via suppressing ERCC1 protein.	[29]
		p38γ expression is down-regulated upon IFN-γ treatment and induced G1/S phase cell cycle arrest.	[31]
Colorectal cancer	Oncogene	Overexpressed p38γ activates the c-Jun, recruits p38γ to MMP-9, leads to the increasing MMP-9 expression and enhances cell invasion.	[34]
		p38γ activates β-catenin/Wnt pathways which promotes CRC development in a colitis-associated mouse model.	[33]
		Overexpression of p38γ promoted CRC cell progression. p38γ is markedly upregulated in colon cancer tissues compared to surrounding colon epithelial cells.	[32]
Pancreatic cancer	Oncogene	p38γ knockout or p38γ inhibitor combination with a PFKFB3 inhibitor suppressed aerobic glycolysis and PDAC tumorigenesis in KPC mice.	[38]
Liver cancer	Oncogene	High p38γ expression was associated with a lower survival rate in liver cancer while p38γ knockdown suppressed proliferation and colony formation in HCC cell lines.	[39]

## p38 in immune regulation

p38α MAPK mediates the production of inflammatory cytokines in different immune cells [41]. p38 enhances transcriptional activity of NF-κB in primary human astrocytes via acetylation of p65 NF-κB [42], a key regulator of the inflammatory response, which contributes to chemoresistance through MDR1 expression in cancer cells [43]. The p38α pathway can promote inflammation in several cell types. Activated myeloid p38α enhances intestinal insulin‐like growth factor‐1 (IGF‐1) production in intestinal inflammation and tumorigenesis [44]. This study showed a significant correlation between p38α phosphorylation in monocytes/macrophages and IGF‐1 phosphorylation in samples from ulcerative colitis patients and colon cancer patients. p38α activation in dendritic cells promotes the expression of proinflammatory cytokines and chemokines and suppresses the expression of anti-inflammatory cytokine in the colon of DSS model, leading to colitis-associated tumorigenesis [45]. On the other hand, p38α also has anti-inflammatory functions in innate immune cells, which are mediated by the mitogen- and stress-activated kinases 1 and 2 (MSK1/2) resulting to the expression of anti-inflammatory genes such as IL-10, DUSP1, TTP, and IL-1ra [46].

## p38 and metastasis

Gamma synuclein (SNCG), a neuronal protein, is overexpressed in different types of cancer. SNCG have been shown to promote TGF-β-induced p38 MAPK phosphorylation by stabilizing MAPK kinase 3/6 (MKK3/6). The upregulation of p38 MAPK by SNCG leads to increased MMP-9 expression, which enhances cancer cell invasion. Overexpression of SNCG in liver cancer cells supports lung metastasis, which can be suppressed by the p38 MAPK inhibitor [47]. IL-1β induces the activation of p38 and the upregulates of MMP-2 and MMP-9 by activating AP-1-dependent transcription in gastric adenocarcinoma (GA) cells. Phospho-p38 is upregulated and correlates with the expression of IL-1β, MMP-2, MMP-9 and c-fos in human GA tissues and in a GA metastasis mouse model. IL-1β also activated JNK but it was not associated with migration and invasion in GA cells [48].

## p38 regulates cellular homeostasis, autophagy, ubiquitination, and proteolysis

### Cellular homeostasis

The p38α pathway plays a dual role during colorectal tumorigenesis [49]. In normal colon epithelial cells, p38α maintains intestinal homeostasis and barrier function to suppress colitis-associated tumor initiation. On the other hand, p38α contributes to colon tumor development by supporting proliferation and inhibiting apoptosis of transformed epithelial cells. TGF-β1 mediates the mRNA and protein levels of MMPs (MMP-2 and MMP-9) and their inhibitors (TIMP-2 and RECK), which plays an essential role of extracellular matrix homeostasis control in breast cancer progression. TGF-β1 phosphorylates p38 MAPK which can induce the expression of MMP-2 and TIMP-2, and increased migration and invasion in breast cancer cells [50].

## Autophagy

Autophagy is a conserved process that recycles damaged cellular proteins, organelles, and other cellular components to maintain energy homeostasis and to protect cells against stress [51]. p38 MAPK have been shown to positively and negatively regulate autophagy. Various stimulations (including oxidative stress, UV, inflammatory cytokines, growth factor, and chemotherapy) can activate p38 MAPK through the TAK1-MKK3/6-p38 and ASK1-MKK3/6-p38 cascades [52]. Next, activated p38 phosphorylates Atg5 leading to inhibition of autophagic membrane extension and the transformation of LCI to LCII, which inhibits the autophagy pathway [53]. p38 can regulate autophagy in response to chemotherapeutic agents. Irinotecan (IRI) induces autophagy and apoptosis through accumulation of reactive oxygen species (ROS) and activation of the JNK and p38 MAPK pathways that leads to tumor suppression in gastric cancer [54]. On the other hand, Isoliquiritigenin (ISL) promotes apoptosis and blocks autophagy through p38 activation that results in cell death and tumor suppression in pancreatic cancer [55]. The combination of ISL and Gemcitabine or 5-FU enhances the inhibition of cell viability compared to single agents.

## Ubiquitination

Ubiquitination, an important post-translational modification in cells, is an ATP-dependent cascade adding ubiquitin, a ubiquitously expressed protein consisting of 76 amino acids, to a substrate protein and inducing the degradation of target protein [56]. Ubiquitin can be attached via 7 lysine residues (K6, K11, K27, K29, K33, K48, and K63) or the first methionine (M1), which regulates various cellular processes including endocytosis of membrane proteins, protein degradation, and DNA repair [57–59]. Activation of protease-activated receptor 1 (PAR1), a G protein–coupled receptor (GPCR) for thrombin and inflammation, induces noncanonical p38 MAPK through autophosphorylation (independence of MKK3/MKK6) via a ubiquitin and TAB1–TAB2–dependent pathway on endosomes [60]. This study is the first one revealing the novel insight of GPCR ubiquitination in mediating the p38 pathway. On the other hand, p38 can phosphorylate Snail, which is a key regulator of epithelial–mesenchymal transition, a major step in tumor metastasis in ovarian cancer [61]. This process enhances Snail stability via suppressed DYRK2-mediated phosphorylation, which is important for GSK3β-dependent Snail phosphorylation and βTrCP-mediated Snail ubiquitination and degradation. Activated p38γ and p38δ eliminate the cancer stem cell properties and tumor initiating ability of non-small cell lung cancer cells via the ubiquitination and degradation of stemness proteins SOX2, OCT4, Nanog, KLF4 and c-MYC through MK2-mediated phosphorylation of Hsp27, an important component of the proteasomal degradation machinery [62].

## Proteolysis

Proteolysis is a fundamental hallmark of cancer as malignant tumors overexpress proteolytic enzymes for invasion, metastasis and angiogenesis including plasminogen activation system (PAS) and the matrix metalloproteinase family (MMPs) [63, 64]. Urokinase plasminogen activator (uPA) is overexpressed in gastric carcinoma cells by enhancing the promoter activity through p38 MAPK signaling [65]. In colon cancer, the transcription factor c-Jun is activated by p38γ, and then recruits p38γ into the matrix metalloproteinase 9 (MMP-9) promoter leading to MMP-9 trans-activation and cell invasion [34]. In gastric cancer, activation of p38 MAPK through IL-1 increases cell invasion in vitro and promotes tumor metastasis in vivo via upregulation of MMP-2 and MMP-9 [48]. WEF, an aqueous extract of Eupatorium fortune in Chinese medicine, blocked PMA-induced p38 and JNK phosphorylation and decreased PMA-induced NF-κB activation. This results to suppress the metastatic properties such as anchorage-independent colony formation, migration and invasion, by downregulating the expression and proteolytic activity of MMP-9 in malignant metastatic cancer [66]. In addition, arsenite, an environmental carcinogen, triggers p38 MAPK activation, and subsequently induced cyclin B1 proteolysis through the ubiquitin–proteasome pathway, which contributes to G2 arrest [67]. Following arsenite exposure, DNA repair may activate p38, promote G2-arrest, cell apoptosis and genome instability.

## p38 contribution to the tumor microenvironment

p38 plays dual role in the tumor microenvironment. In breast cancer, tumor-derived GM-CSF induced myeloid cells ARG1 expression through p38 activation and inhibited antitumor function of T cells. ARG1 is a biomarker for protumor M2-polarized macrophages. This results in an immunosuppressive tumor microenvironment which causes resistance to adoptive T cell transfer [48]. On the other hand, AMP-activated protein kinase (AMPK) activates p38 MAPK and phosphorylates glycogen synthase kinase-3β (GSK-3β). This leads to inhibition of PD-1 expression in Tregs and suppresses tumor progression [68]. In addition, CD4 + T cells with activated p38 signaling can promote pancreatic cancer progression [69].

## Targeting p38 gamma for cancer treatment

Overall, there remains some controversy as to the role of p38γ in tumorigenesis and as a target for therapy in gastrointestinal cancers. In general, p38γ levels are overexpressed in many human malignancies including breast cancer [70], gliomas [71], and gastrointestinal cancers [28, 32, 38, 39]. This section will focus on targeting p38 as an oncogene in cancer therapy.

The four p38 MAPK isoforms have different sensitivity to kinase inhibitors. The p38γ and p38δ isoforms (~75% identity) are less similar in sequence compared to p38α (~60% identity) [72]. For instance, pharmacological studies showed that specific compounds (SB203580 and other pyridinyl imidazoles) can only inhibit p38α and p38β, but not p38γ and p38δ [73]. Three main p38α MAPK inhibitors have been in clinical trials: MW150 (by ADDF, in safety studies), Neflamapimod (by EIP Pharma, in efficacy studies [74]) for Alzheimer’s disease and Losmapimod (by GlaxoSmithKline) for myocardial infarction [75]. PH-797804 is an oral p38 inhibitor tested for Rheumatoid Arthritis (Clinical trial NCT00383188). There are some clinical trials targeting p38 for cancer treatment. Ralimetinib (or LY2228820), a selective inhibitor of p38α and p38β, is being tested as monotherapy or in combination with other agents, for the treatment of ovarian cancer [76], glioblastoma, and metastatic breast cancer. This clinical trial (NCT01663857) evaluated the efficacy of ralimetinib in combination with gemcitabine and carboplatin for patients with recurrent platinum-sensitive epithelial ovarian cancer. The combination therapy resulted in the improvement of progression-free survival (PFS). p38 MAPK Inhibitor LY3007113 is being tested for advanced or metastatic cancer [77]. Talmapimod (SCIO-469), an orally active, selective, and ATP-competitive p38α inhibitor, is being tested as a monotherapy or in combination with Bortezomib for relapsed multiple myeloma (Clinical trial NCT00087867) (Table 2).

**Table 2:** p38 inhibitors in clinical trials.

Inhibitor	Target	Disease	Studies
MW150	p38α	Alzheimer	NCT05194163: safety study (phase 2)
Neflamapimod	p38α	Alzheimer	NCT03402659: efficacy study (phase 2) [74]
		Dementia With Lewy Bodies	NCT04001517: efficacy study (phase 2)
Losmapimod	p38α and p38β	Myocardial infarction	NCT00910962: safety and efficacy study (phase 2) [75]
PH-797804	p38α	Rheumatoid Arthritis	NCT00383188: safety and efficacy study (phase 2)
Ralimetinib (LY2228820)	p38α and p38β	Ovarian cancer	NCT01663857: in combination with gemcitabine [76]
		Advanced cancer	NCT01393990
		Metastatic breast cancer	NCT02322853
		Glioblastoma	NCT02364206: in combination with Temozolomide and radiotherapy
LY3007113	p38α	Advanced cancer	NCT01463631: safety study (phase 1) [77]
Talmapimod (SCIO-469)	p38α and p38β	Multiple myeloma	NCT00087867: monotherapy or in combination with Bortezomib (phase 2)

The p38 MAPK inhibitor (SB203580) significantly increased the sensitivity of colorectal cancer cell to 5-FU, a common therapy for colon cancer. The combination of SB203580 and 5-FU markedly reduced cell viability through a decrease of pro-apoptotic protein Bax expression [78]. However, long-term treatment of 5-FU results in chemoresistance. The development of multidrug resistance (MDR) related to the overexpression of ATP-binding cassette (ABC) transporters decreases drug accumulation in cancer cells and cause chemoresistance. The SW480/5-FU cells showed a significantly increased protein expression level of MDR-related proteins (P-gp, MRP1 and ABCG2). Noscapine treatment decreased the expression of these proteins in the SW480/5-FU cells, and combination with p38 MAPK inhibition enhances the sensitivity of 5-FU-resistant colon cancer cells to noscapine [79].

Until now, there is one p38γ inhibitor named pirfenidone (PFD) that suppressed pro-inflammatory cytokines and tumor growth in a colitis-associated CRC model [33] and pancreatic cancer mouse model [38]. Pirfenidone has been clinically tested for patients with idiopathic pulmonary fibrosis [80]. A combined p38γ and p38δ inhibitor (BIRB796) reduced IFN-γ [81]. A potent p38γ inhibitor (F7 or PIK75) effectively suppressed tumor growth in a cutaneous T-cell lymphoma (CTCL) mouse model [82]. This small molecule p38γ inhibitor has been screened through a high-throughput kinase inhibitor library. F7 or PIK75 inhibited p38γ kinase activity, significantly reduced tumors burden in mice, and eliminated CD4+ malignant CTCL cells but not healthy CD4+ cells. Based on the Drugbank platform, several p38γ-targeted drugs including phosphonothreonine, phosphoamino phosphonicacid-adenylate ester, CEP-1347 and KC706 are being investigated and tested [83, 84].

In the past 15 years immnunotherapy has evolved as a therapeutic strategy for several solid tumors. Different types of immunotherapies have been investigated in GI cancers, including adoptive T-cell transfer [85, 86], dendritic cell vaccines [87, 88], peptide vaccines [89, 90], and immune checkpoint inhibitors [91–94].

Recently, using CRISPR-Cas9 targeted 25 TCR-driven kinases, Gurusamy et al. found that knockout MAPK14 (p38α) can increase T cell expansion and memory. This also decreases reactive oxidative stress (ROS), and genomic stress (gH2AX) which accounts for an effective anti-tumor T cell [95]. Anti-CD19 CAR T cells expanded with the p38 inhibitor (BIRB796) markedly suppressed tumor growth and enhanced survival in tumor bearing mice. Therefore, it is interesting to combine p38 inhibitors and other immunotherapies like adoptive T cell transfer to enhance therapeutic effects in GI cancers.

## Conclusion

**Fig. 3:**
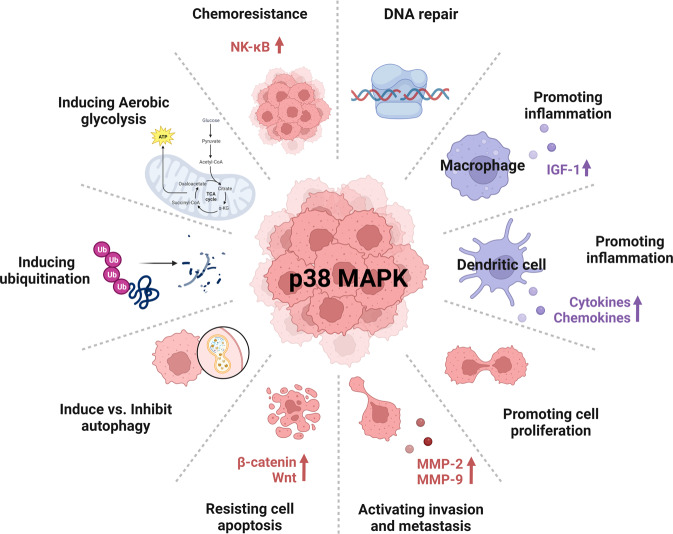
p38 in cancer hallmarks and immune response.

p38 MAPKs have been explored as regulators of environmental stress and inflammation as well as mediators of homeostasis maintenance. Recent studies implicate crucial functions of p38α in the tumorigenesis and cancer development. In this review, we summarized the primary impact of p38 in gastrointestinal cancers, in which p38 is overexpressed, plays as an oncogene through regulation of various cellular processes (metastasis, autophagy, ubiquitylation and proteolysis) and facilitating tumor malignancy (Fig. 3). Future research work will clarify which isoform of p38 is more important and valuable for combination therapy in gastrointestinal cancers. An in-depth understanding of the p38 pathway and downstream mechanisms will translate into better therapeutic strategies.
